# Evaluating the effect of enzymatic pretreatment on the anaerobic digestibility of pulp and paper biosludge

**DOI:** 10.1016/j.btre.2017.12.009

**Published:** 2018-01-05

**Authors:** Sofia Bonilla, Zahra Choolaei, Torsten Meyer, Elizabeth A. Edwards, Alexander F. Yakunin, D. Grant Allen

**Affiliations:** Department of Chemical Engineering and Applied Chemistry at the University of Toronto, 200 College St., Toronto, Ontario M5S 3E5, Canada

**Keywords:** Anaerobic digestion, Biosludge, Enzymes, Pretreatment, Biogas

## Abstract

Anaerobic digestion of biosludge has not yet been implemented in pulp mills due to low biogas yields. Enzymatic pretreatment of biosludge has shown improvements in biogas yields but results are varied. A key limitation of previous studies is that they fail to consider the COD contribution from the enzyme solutions. The aim of this study was to systematically investigate the potential for enzymatic pretreatment on the anaerobic digestibility of pulp mill biosludge. Out of the six enzymes tested, four enhanced the anaerobic digestibility of biosludge. At the end of the BMP, a maximum improvement of 26% in biogas yield was observed with protease from *B. licheniformis*. There was no correlation between enzymatic activities on standard substrates and/or on biosludge and the effect of enzymes on biogas yields. Enzymes have potential for improving biosludge anaerobic digestibility but more research on optimal conditions and potential synergies with other pretreatment is needed.

## Highlights

• A new and rigorous approach for assessing the effect of enzymatic pretreatment on AD is proposed.
• Enzymes can improve anaerobic digestion increasing biogas yields by up to 26%.
• First study to isolate the effect of catalytic activity and organic load from enzymes to evaluate enzymatic pretreatment.
• Enzymes did not appear to be inhibited or denatured in the presence of biosludge.

## Graphical abstract

**Figure:**
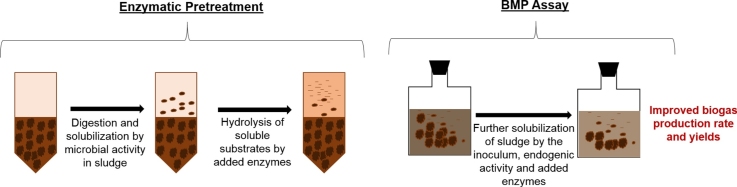

## 1 Introduction

There is increasing interest in developing technologies to reduce biomass produced during wastewater treatment processes in pulp and paper (P&P) mills. Sludge management accounts for up to 60% of treatment costs [1]. Anaerobic digestion of sludge is extensively used in municipal wastewater treatment but its implementation in pulp mills is still limited. The mass and volume reduction afforded by anaerobic digestion translates into savings associated with sludge handling and disposal, and the recovery of energy from biogas make this a very attractive process. However, the use of anaerobic digestion for P&P mill biosludge has not been industrially established because of low methane yields, reportedly due to the complexity and recalcitrance of pulp and paper mill biosludge and the presence of toxic chemicals [2]. As discussed in recent reviews by Elliott & Mahmood (2007) and Meyer & Edwards (2014), several biosludge pretreatment approaches have been investigated for improving the feasibility of anaerobic digestion of biosludge in P&P mills [[2], [3]].

Enzymatic pretreatment of biosludge can potentially enhance methane yields. Hydrolysis is widely accepted as the limiting step in the anaerobic conversion of the complex organic matter in biosludge. Enzymes that can speed-up hydrolysis are gaining attention because of their catalytic activity and potential to be produced from renewable and/or waste sources [4]. Discovery of novel enzymes, enzyme engineering, and the reduction of production costs is driving the development of many enzyme-based technologies. As discussed in Parawira (2012), enzymes are recognized for their potential to hydrolyze biosludge, resulting in improved anaerobic digestion. However, the effects of enzymatic pretreatment are poorly understood [5]. To date, studies have concentrated primarily on municipal biosludge with conflicting findings. While some authors have reported a substantial improvement in biogas production, methane yield, and/or chemical oxygen demand (COD) solubilization [[6], [7], [8], [9]], others found improvements only in lab-scale experiments but not in pilot scale [10] and still others found no improvement [11].

Proteases and glycosidases are the obvious first enzyme candidates for pretreatment, because biosludge is mainly composed of microbial biomass comprising proteins and complex carbohydrates. In addition, the particles in biosludge are embedded in a gel-like matrix of extracellular polymeric substances (EPS) comprising different biopolymers, including proteins, carbohydrates, lignin, DNA, and RNA [[12], [13]]. Proteins and carbohydrates account for up to 70% of the organic matter present in P&P biosludge [2]. Accordingly, previous studies employed proteases, glycosidases, or a combination thereof for biosludge treatment [[6], [9], [10], [11]].

In reviewing the work done in previous studies on the enzymatic pretreatment of biosludge we identified three possible confounding factors, which we addressed in this study. First, in only two studies does the chemical oxygen demand (COD) contributed by the enzymes appear to be taken into account [[10], [11]]. Second, enzymes are polypeptides and, as polymers, could have an effect on biosludge flocculation and possibly digestibility that is not related to their enzymatic activity. For example, proteins can induce flocculation of sludge particles as has been reported previously [14]. Accordingly, the use of inactivated enzyme controls is needed to confirm a catalytic mechanism to a pretreatment. Finally, deconvoluting biogas produced from biosludge from biogas produced by digestion of the inoculum is important to quantify the effect of enzymes on biogas yield from the intended substrate (biosludge). Reducing the “background” biological activity of the system (i.e. biogas from inoculum) enables quantification of the true impact of the enzymatic pretreatment on biosludge. Addressing these issues will lead to a more accurate assessment of the potential of enzymatic pretreatment for enhanced anaerobic digestibility of biosludge at larger scale. With these considerations, the specific objectives of this study were:

• To develop an experimental methodology that evaluates the effect of enzymatic pretreatment on anaerobic digestibility separately from any effects related to the enzymes as organic additives.
• To test hydrolytic enzymes from two groups, proteases and glycosidases, for their potential to enhance the anaerobic digestibility of biosludge.
• To measure enzymatic activity using standard substrates added into biosludge, to detect possible inhibitions or synergies.
• To measure changes in soluble COD content during enzymatic pretreatment of biosludge to better characterize the process.

## 2 Materials and methods

**Fig. 1:**
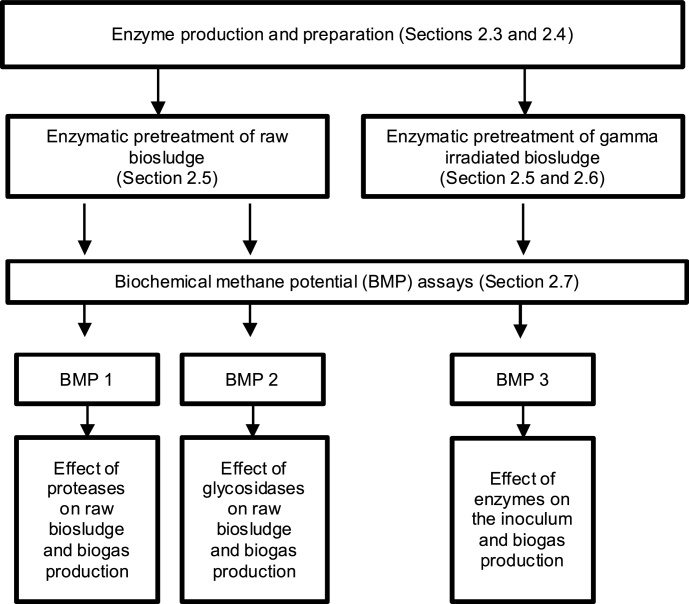
General approach for investigating the effect of enzymatic pretreatment on biosludge anaerobic digestibility.

The approach used to meet these objectives involved three biochemical methane potential (BMP) assays, enzymatic and compositional analyses. A flow diagram of the general approach is provided in Fig. 1.

### 2.1 Biosludge samples

Waste activated sludge, or biosludge, from a secondary clarifier was obtained from a Canadian P&P mill that produces a variety of pulp, paper and specialty products using sulfite pulping and mechanical pulping (bleached chemi-thermomechanical pulp − BCTMP). Samples were kept at 4 °C in the laboratory prior to the experiments and for a maximum of two weeks. Before use in experiments, biosludge samples were allowed to settle overnight in a cold room at 4 °C, and the supernatant was discarded to obtain a thickened sludge.

Gamma irradiated biosludge was used in one experiment (BMP 3) to inactivate microorganisms in the biosludge to enable testing enzyme activity without confounding effects from microbial activity inherent to biosludge. Sludge was irradiated at a dose of 25 kGy produced from a cobalt source (Co-60) using a Gamma Cell (G.C. 220). Previous studies have reported a > 99% inactivation of common pathogens present in sewage sludge at a dose of 5 kGy [15].

### 2.2 Anaerobic inoculum (Granules) preparation

Anaerobic granules were used as the inoculum for the biochemical methane potential (BMP) assays described in section 2.6. Granules were obtained from the anaerobic wastewater treatment reactor of a Canadian pulp and paper mill and were maintained in the laboratory under anaerobic conditions at 4 °C. Two weeks before the BMP set up, anaerobic granules were diluted (1:2) in a synthetic medium described in [16]. The diluted granules suspension was then incubated at 37 °C and fed with the synthetic feed (0.4% v/v) previously reported by [17]. The anaerobic activity of the inoculum was confirmed by measuring biogas production. The inoculum was left incubating until the day of the experiment. This two-week incubation period reduced the easily digestible COD, minimizing the background biogas produced in the BMP assays.

### 2.3 Enzyme preparations

The enzymes used in this study were hydrolases from two subgroups: proteases (EC 3.4) and glucosidases (EC 3.2.1). A preliminary screening of proteases and glucosidases was conducted in our laboratory. Based on their activity on standard substrates six enzymes were selected. Four of the enzymes were available commercially and two were produced in our laboratory. General information about the enzymes used in this study is presented in Table 1.

**Table 1:** General information of enzymes used in this study.

Enzymes	EC Number	Activities	Source
Protease from *Bacillus licheniformis*	3.4.21.62	Serine protease (subtilisin)	Sigma-Aldrich (P4860)
Protease from *Aspergillus oryzae*	3.4.-	Mixture of seven peptidases and one α-amylase^a^	Sigma-Aldrich (P6110)
BCE_2078 from *Bacillus cereus* (Q739R2)	3.4.21.-	Serine protease	Produced in-house
Lysozyme from chicken egg white	3.2.1.17	Glycosidase	Bioshop (LYS702)
Cellic^®^ CTec 2	3.2.1.-	Mixture of cellobiohydrolase I, endoglucanase, and β-glucosidase^b^	Novozymes
SCO6604 *from Streptomyces coelicolor* (Q8CJM3)	3.2.1.21	β-glucosidase	Produced in-house

a Merz et al. [18].

b Rodrigues et al. [19].

#### 2.3.1 Commercial enzymes preparation

Solutions of commercial enzymes (25% v/v) were prepared in 50 mM phosphate buffer at pH 7. The solutions were then dialysed overnight against the same buffer using a Pur-A-Lyzer™ Mega Dialysis Kit (Sigma-Aldrich, St. Louis, USA). After dialysis, half of the enzyme solution was taken to prepare the inactive enzyme solution by placing it in an oven at 103 °C for 6 h followed by immediate exposure to −20 °C for at least 2 h. This temperature shock resulted in the irreversible inactivation of the enzyme as verified in the enzymatic assays described in section 2.4.

#### 2.3.2 Cloning, overexpression and purification of novel enzymes

The production of novel enzymes was carried out as described by Gonzalez *et al. (*2006), with a few modifications [20]. The recombinant plasmid (p15TvL) containing the coding gene for the His-tagged proteins (BCE_2078 or SCO6604) was transformed into *Escherichia coli* strains (BL21) for overexpression. Cells were grown in terrific broth (TB) to an OD_600_ of approximately 1 and protein expression was induced with 0.4 mM isopropyl-d-thiogalactopyranoside. After induction cells were incubated overnight at 16° C. The harvested cells were re-suspended in buffer A (50 mM HEPES, pH 7.5, 5 mM imidazole and 5% v/v glycerol) and sonicated. The cell debris was then pelleted by centrifugation at 21,000*g* for 45 min in a Beckman-coulter centrifuge (Avanti JE, rotor JLA 16.250). BCE_2078 and SCO6604 were affinity purified from the soluble fraction using Ni-NTA resin (Qiagen, Hilden, Germany), followed by washing the column with buffer B (same as buffer A but 50 mM imidazole) and eluting with buffer C (same as buffer A but 250 mM imidazole). SDS-gel electrophoresis was used to verify the purification of the enzyme of interest. The eluted enzymes were dialysed overnight and further processed as described in section 2.3.1 of this document.

### 2.4 Enzymatic assays

Assays were conducted to confirm the activity of the enzymes prior to biosludge treatment and, to potentially correlate these enzymatic activities to the effect of enzymatic pretreatment on biosludge anaerobic digestibility. For proteases and glycosidases (except lysozyme), assays with standard substrates, biosludge, and a combination thereof, were used to evaluate enzymatic inhibition by biosludge. Lysozyme’s activity on biosludge could not be measured because biosludge interferes with the basis of the lysozyme activity assay (i.e. cell optical density). The specific details of the enzymatic assays are described in Section 2.4.1 and 2.4.2.

#### 2.4.1 Protease activity

A modified version of Sigma’s protease activity assay (Sigma Aldrich, Universal Protease Activity Assay: Casein as a Substrate) was used for the detection of protease activity. In 96-well plates, 200 μg of enzyme was incubated with 25 μL of a 40 g/L casein (standard substrate) solution at 37 °C for 30 min, final volume of all wells was maintained at 185 μL. The reaction was stopped by adding 185 μL of a trichloroacetic acid (TCA) solution (20% w/w) and incubated at 37 °C for 30 min. Plates were centrifuged at 13,000 rpm (Eppendorf centrifuge 5417C) and the supernatant was recovered. For colorimetric detection, sodium carbonate (310 mM) was added to 88 μL of the supernatant followed by the addition of 60 mM Folin-Ciocalteau phenol reagent, in 96-well plates. After 30 min of incubation at 37 °C, the absorbance was read at 660 nm. Blanks were prepared by adding the TCA before enzyme addition. In addition to casein as the standard substrate, samples with biosludge only, and biosludge plus casein were used to assess enzymatic activity, potential synergies and inhibitions. Protease activity is presented as mM tyrosine equivalents released per g of enzyme per min (mM Tyr/g enzyme/min) using a tyrosine calibration curve. All assays were carried out in triplicate for active and inactive proteases.

#### 2.4.2 Glycosidase activity

Glycosidase activity assays were conducted based on the use of dinitrosalicylic (DNS) acid reagent for the measurement of reducing glucose. The DNS reagent was prepared by dissolving 5 g of 3,5-dinitrosalicylic acid in 200 mL of ddH_2_O while heating at around 50 °C. To this solution, 50 mL of 4 N sodium hydroxide and 150 g sodium potassium tartrate were added, and the volume was adjusted to 500 mL. The assay was started by incubating 200 μg of enzyme with 1% carboxymethyl cellulose (CMC) in 96 well plates, at a total volume of 200 μL, for 1 h at 37 °C. One volume of this sample was mixed with one volume of DNS reagent, and incubated at 100 °C for 10 min. Afterwards, the plate was cooled down at room temperature, and the absorbance was recorded at 540 nm against a blank (containing phosphate buffer instead of enzyme solution). As with proteases, samples with biosludge only, and biosludge plus CMC, were used to assess enzymatic activity on biosludge, potential synergies and inhibitions. Glucose concentration was calculated using a glucose standard curve. All assays were carried out in triplicate for active and inactive enzymes.

Lysozyme activity was measured using Sigma’s lysozyme assay (Sigma Aldrich, Enzymatic Assay of Lysozyme). A suspension containing *Micrococcus lysodeikticus* (0.01% w/v) purchased from the same company in potassium phosphate monobasic (66 mM, pH 6.2) was prepared. In cuvettes with 1 mL of the *M. lysodeikticus* cell suspension, the absorbance was measured and used as the blank. Lysozyme solution was added (0.1 mL) and the change in absorbance was monitored overtime for 5 min. All assays were carried out in triplicate for active and inactive enzyme. However, for lysozyme, assays on biosludge or biosludge and cells could not be performed since the assay used the absorbance of cells and no distinction could be made between cells from biosludge and cells from *M. lysodeikticus.*

### 2.5 Biosludge pretreatment

Solutions of active and inactive enzymes were added to the thickened biosludge and incubated for 6 h at 37 °C and shaken using an orbital shaker incubator (Amerex Gyromax 747R) at 100 rpm. Final enzyme concentrations were adjusted to 1% (protein/TSS biosludge). Protein concentrations were measured using the Bradford Reagent (Biorad, California, USA), where bovine serum albumin (BSA) calibration curve was used to determine the amount of enzyme solution to be added. Biosludge with deionized water and biosludge with phosphate buffer were used as controls in order to account for the potential effects of buffer in the system. The volume of biosludge, enzymes, water or buffer was maintained constant for all the samples. At the end of the incubation period, the final COD concentration was used to calculate the amount of biosludge to be added to the BMP assays. For BMP 3, the enzymatic pretreatment was carried out for 24 h instead of 6 h to measure the effect of enzymatic treatment over a longer period of time. Chemical analyses were carried out on samples taken at 0, 4, 7 and 24 h. Five ml of active or inactive enzyme solutions were added to 45 mL of the thickened biosludge and incubated for 6 h at 37 °C and shaken using an orbital shaker incubator (Amerex Gyromax 747R) at 100 rpm. Final enzyme concentrations were adjusted to 1% (protein/TSS biosludge). Protein concentrations were measured using the Bradford Reagent (Biorad, California, USA), where a bovine serum albumin (BSA) calibration curve was used to determine the amount of enzyme solution to be added. Biosludge with deionized water and biosludge with phosphate buffer were used as controls in order to account for the potential effects of buffer in the system. The volume of biosludge, enzymes, water or buffer was maintained constant for all the samples. At the end of the incubation period, the final COD concentration was used to calculate the amount of biosludge to be added to the BMP assays. For BMP 3, the enzymatic pretreatment was carried out for 24 h instead of 6 h to measure the effect of enzymatic treatment over a longer period of time. Chemical analyses were carried out on samples taken at 0, 4, 7 and 24 h.

### 2.6 Chemical analyses

#### 2.6.1 Total and suspended solid analyses

Total suspended solids (TSS) and volatile suspended solids (VSS) for biosludge and anaerobic granules samples were quantified according to the Standard Methods for the Examination of Water and Wastewater [21].

#### 2.6.2 Chemical oxygen demand (COD)

Total chemical oxygen demand (tCOD) was analysed following the Standard Methods for the Examination of Water and Wastewater [21]. For soluble chemical oxygen demand (sCOD) measurements, the samples were first centrifuged at 13,000 rpm for 10 min using an Eppendorf microcentrifuge (5417C). In this study, the supernatant was considered as the soluble fraction and was further analysed using TNTplus™ vials, Hach Method 8000 with range 3–150 mg/L COD (Hach Co., USA).

### 2.7 Biochemical methane potential (BMP) assays

The biochemical methane potential (BMP) assays first described in [22] were modified to evaluate the anaerobic digestibility of enzymatically-pretreated biosludge. The assays were prepared in a disposable anaerobic glove bag with Zipper-lock closure (Sigma-Aldrich, St. Louis, USA) filled with a gas mixture with the composition of 80% N_2_, 10% CO_2_, and 10% H_2_ by volume. All samples were prepared in triplicates in 160 mL serum bottles. In each set of experiments, all bottles contained the same volume of anaerobic granules (10 mL) and synthetic anaerobic medium (60 mL). The volume of biosludge added was adjusted to maintain the same COD in all the bottles, biosludge added ranged 8.1–9.2 mL in BMP 1, 8.8–12.4 mL in BMP 2 and 8.7–10 mL in BMP 3. The liquid volume was maintained at 80 mL using deionized, sterile, anaerobic water. Once prepared, bottles were incubated at 37° C and 100 rpm for at least 60 days.

Inoculum to substrate ratios (ISRs) used in this study were 0.4 and 0.8 based on total COD, equivalent to 0.4 and 1.0 based on volatile solids (VS) (see Table 2). It has been previously reported that ISRs affect the rate of anaerobic digestion and if the ISR is <0.5 (VS basis), acidification due to volatile fatty acids accumulation may delay or inhibit methane production [[23], [24]]. However, for the purpose of this study, high ISRs ratios are not advisable because they result in large amounts of biogas produced from the inoculum (compared to the biogas produced from the actual samples of interest, i.e. biosludge). This hinders the ability to assess the effect of different enzymes. Potential effects from the ISRs used in this study were also considered.

**Table 2:** Characteristics of raw biosludge, inoculum, and inoculum-to-substrate ratios based on COD used in the three biochemical methane potential (BMP) assays performed in this study.

	BMP 1 (Feb 02/2015)	BMP 2 (Mar 3/2015)	BMP 3^a^ (Jul 07/2015)
Raw biosludge characteristics			
Biosludge (3 distinct samples for each BMP)	Sample 1	Sample 2	Sample 3
TSS (g/L)	18.8 (±0.7)	18.6 (±0.3)	20.1 (±0.8)
VSS (g/L)	16.1 (±0.6)	15.8(±0.1)	17.6 (±0.8)
COD (g/L)	24.2 (±0.4)	25.2 (±1.8)	27.8 (±2.8)
Granules (3 distinct samples for each BMP)			
TSS (g/L)	17.7 (±0.2)	26.1(±1.3)	19.9 (±1.7)
VSS (g/L)	15.6 (±0.1)	25.6 (±1.1)	17.9 (±1.4)
COD (g/L)	26.2 (±1.4)	33.1 (±1.3)	21.6 (±2.4)
COD contribution in BMP bottles (mg COD/bottle)			
Granules (inoculum)	86	122	151
Biosludge (substrate)^b^	200	150	200
Inoculum to substrate ratio^c^	0.4	0.8	0.8

a Biosludge in BMP 3 was gamma irradiated.

b COD was measured after enzymatic treatment.

c Ratio was calculated based on COD.

Controls were added to BMP assays to investigate the effect of inactive enzymes, biosludge and granules on biogas yields. In addition, in each assay, the synthetic feed used in section 2.2 was used instead of biosludge, maintaining the same COD/bottle, to evaluate the methanogenic activity of the granules with easily digestible substrates (i.e. a mix of glucose, sodium acetate, sodium propionate and methanol), these samples will be referred throughout this document as “positive controls”. Samples named “inoculum only” were used as the experimental blank, they represent the background methanogenic activity from the inoculum. When the biogas and the specific biogas yield (SBY) are reported, the amount of biogas produced from these inoculum-only bottles, is subtracted from all the samples that contained inoculum (see Eq. (1)). Samples named “biosludge only”, i.e. without the inoculum, were used to evaluate the self-digestibility of biosludge. BMP assays were also carried out on biosludge pre-treated with inactive enzymes to account for COD contributions from the enzymes themselves. To evaluate the digestibility and gas production from the enzyme solutions specifically, BMP 3 included bottles where enzyme solutions were added with inoculum and synthetic medium (without biosludge).

#### 2.7.1 Biogas production

Biogas production was measured using a water-lubricated glass syringe [22]. Since the BMP assays were prepared in a glove bag at room temperature, and then sealed bottles were moved to an incubator at 37° C, initial biogas samples will include the volume of gas associated with expansion caused by the increase in temperature. To correct for this effect, the amount of biogas produced after 24 h in the negative controls (biosludge only) was subtracted from all the samples at that time point. For data analysis and treatment comparison, both specific biogas yield (SBY) and total biogas production (TBP) were computed, as per Eqs. (1) and (2) below (see Supplementary data):

(1) $$SpecificBiogasYield\left(SBY\right)\left(mLg^{-1}COD\right)=\frac{CumulativeBiogas{\;}_{sample}\left(mL\right)-CumulativeBiogas{\;}_{inoculum}\left(mL\right)}{COD_{substrate}\left(g\right)}$$

Where COD_substrate_ is the COD added from the biosludge sample. The COD was measured after enzymatic treatment. Specific biogas yield represents the final BMP yield. Thus, cumulative biogas at the end of each BMP were used.

(2) $$TotalBiogasProduction\left(TBP\right)\left(mLg^{-1}COD\right)=\frac{Biogas_{sample}\left(mL\right)}{COD_{substrate}\left(g\right)+COD_{inoculum}\left(g\right)}$$

In addition, the theoretical biogas potential was calculated and used as a benchmark for complete conversion of organic matter to methane and carbon dioxide. This full conversion is expected for the synthetic feed which is composed of easily digestible compounds [17]. Using the equivalence of 1 g COD to 397 mL CH_4_ at 37 ° C [25] and the CH_4_ concentration in biogas, the theoretical biogas production was calculated with Eq. (3):

(3) $$TheoreticalBiogasPotential\left(ml\right)=\frac{CODadded\left(g\right)x397CH_{4}\left(mL/gCOD\right)}{CH_{4}concentration\left(\%\right)}$$

#### 2.7.2 Methane analysis

Using a 500 μL glass-tight syringe, 200 μL of the headspace were removed and injected into a Hewlett Packard 5890 equipped with CTR I packed column and a thermal conductivity detector (TCD). The column head pressure was maintained at 22–24 psi with Helium as the carrier gas. The oven temperature was isothermal at 50° C. The injector and detector temperature was 200° C for both. Methane standards were used to prepare a calibration curve and methane eluted at 8.3 min. Methane production was calculated for every sampling day (see Supplementary data) using the following equation:

(4) $$MethaneProduced\left(mL\right)=\frac{Biogasproduced\left(mL\right)xmethaneconcentrationfromGC\left(\%\right)}{100}$$

## 3 Results and discussion

### 3.1 Set up conditions of BMP assays

Biosludge and anaerobic granules used in the three BMPs conducted in this study were collected at different times in the mill and used at different times in the laboratory; thus, there is variability in their COD, VSS, and TSS composition (Table 2). Given the conditions of each BMP, inoculum to substrate ratios were different and defined based on the COD content as shown in Table 2.

### 3.2 Effect of enzymatic pretreatment of biosludge on biogas production

**Fig. 2:**
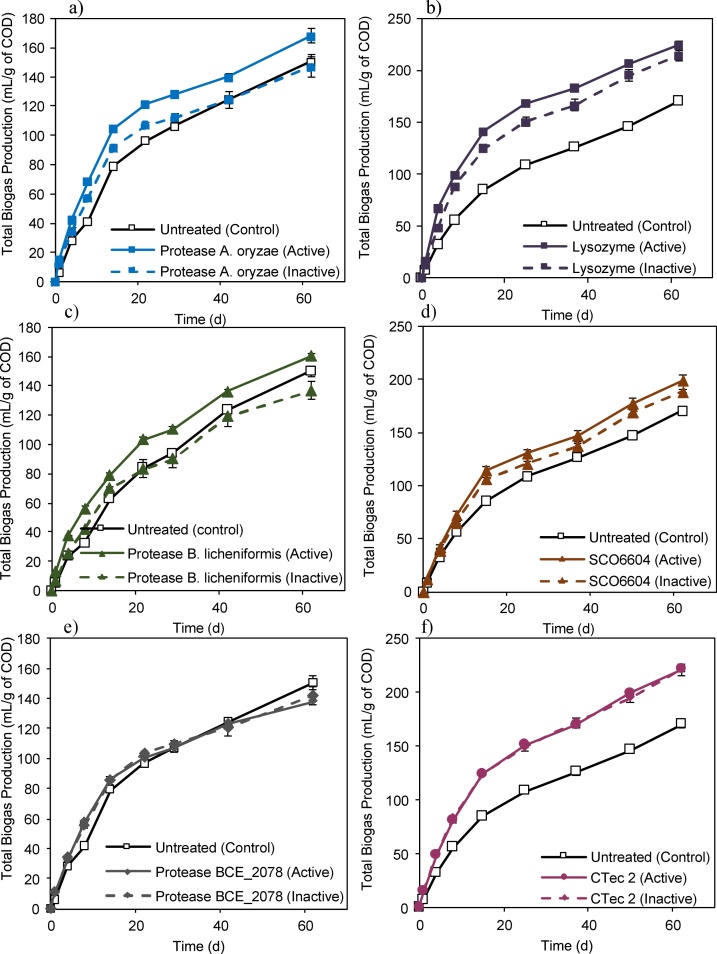
Specific biogas production, SBP, of biosludge pretreated with enzymes over the 62 days of anaerobic digestion. a) protease from *A. oryzae*; b) lysozyme; c) protease from *B. licheniformis*; d) glycosidase SCO6604; e) protease BCE_2078 and f) CTec 2. Untreated (control) for all samples had phosphate buffer added to biosludge instead of enzyme solution. Range differences between BMP 1 (a, c, e) and BMP 2 (b, d, f) are mainly due to differences in biosludge and granules used in each BMP, inoculum to substrate ratios and, soluble chemical oxygen demand (sCOD) variations.

The addition of certain proteases and glycosidases lead to a significant increase in biogas production in comparison to the untreated biosludge control (Fig. 2). The proteases from *B. licheniformis* and *A. oryzae*, the glycosidase SCO6604 and lysozyme showed improvements over their inactivated controls, while other enzymes, (BCE_2078 and CTec 2) did not. In these experiments, biogas may originate from digestion of the soluble COD added in the enzyme solution and from enhanced hydrolysis and digestion of the organic matter in biosludge. To make sure that any extra biogas comes from biosludge and not enzymes solution, we compared active to inactivated enzyme to accurately measure the effect of enzymatic hydrolysis alone.

Our results revealed that the activity of specific proteases and glycosidases tested did improve the anaerobic digestibility of biosludge, thus demonstrating that enzymatic pretreatment can contribute to more gas production. Not all of the enzymes tested improved digestibility, and thus it is clear that the success of the enzymatic pretreatment is a function of enzyme type, enzyme stability, dose, incubation conditions, inhibitors, and many other factors. Further testing is needed to identify the key factors and optimize pretreatment strategy for a given waste type. However, to maximize pretreatment effect, it is first imperative that the assay used to evaluate alternatives be sufficiently accurate and discriminating. Several confounding factors can mask real effects or mislead interpretation of results. These include inoculum reproducibility from test to test, effects related to varying inoculum to substrate ratio, biogas production from the enzyme solutions, and errors relating to COD measurement in heterogeneous systems. This study allowed for analysis of these effects, as described below.

### 3.3 Effect of inoculum, substrate and ISR on biogas composition and biogas production

Biogas production is known to be affected by inoculum to substrate ratios (ISR), as well as biosludge and inoculum quality. As can be seen in Table 3, yields (SBP and TBP) for the controls used in this study were different in each experiment, that may relate back to differences in ISRs. Biogas production yields were generally lower in the BMP test with the lowest ISR (0.4). The activity of the inoculum and the particular biosludge sample used also play a role. Therefore, comparisons within a BMP test that uses the same conditions, including all relevant controls, can be made, for example as we did in this study where enzyme pretreated samples were compared against individual controls (biosludge without enzymatic treatment and inoculum) or biosludge with inactive enzyme and inoculum. However, comparisons between BMP assays must be made with caution given that the inoculum (i.e. granules), substrate (i.e. biosludge) and/or ISRs are different. In addition, although the background biogas produced by the inoculum should be minimized, using a low ISR such as 0.4 may hinder the biogas production obtained during the BMP, as it is shown in Table 3, where the theoretical maximum was not achieved even after 62 days of digestion. An ISR of 0.8 has been shown (See Table 3) to sufficiently reduce background biogas production while allowing maximum biogas production rate. Other differences between BMP assays are due to the variability in the composition and structure of biosludge and granules between batches. Effect of enzymatic treatment of biosludge on soluble COD.

**Table 3:** Effect inoculum-to-substrate ratio (ISR) on total biogas production (TBP), specific biogas yields (SBY) and methane concentration

Sample	ISR	TBP (ml/g COD total)^a^	SBY (ml/g COD fed)^b^	Methane Concentration (%)
BMP1 Inoculum only	0.4	153 (±6)	N/A	76
BMP2 Inoculum only	0.8	103 (±3)	N/A	69
BMP3 Inoculum only	0.8	168 (±19)	N/A	80
BMP1 Biosludge + Inoculum	0.4	150 (±3)	148 (±3)	74
BMP2 Biosludge + Inoculum	0.8	171 (±4)	225 (±9)	75
BMP3 Biosludge (gamma irradiated) + Inoculum	0.8	164 (±16)	150 (±9)	75
BMP1Synthetic feed + Inoculum (Positive Control)^c^	0.4	344 (±8)	425 (±11)	74
BMP2 Synthetic feed + Inoculum (Positive Control)^c^	0.8	316 (±8)	489 (±15)	75
BMP3 Synthetic feed + Inoculum (Positive Control)^c^	0.8	340 (±12)	462 (±16)	87

All values were calculated base on the last biogas sample of each BMP assay i.e. 62 days of anaerobic digestion for BMP 1 and 2 and 50 days for BMP 3.

a Biogas produced per total chemical oxygen demand (COD) in the bottle.

b Biogas produced per chemical oxygen demand (COD) fed. Biogas produced from the inoculum was subtracted from all samples with inoculum.

c For reference, the theoretical maximum biogas production for the synthetic feed is 532 (±35) ml/g COD fed and methane content in the biogas should be between 70 and 80%).

**Fig. 3:**
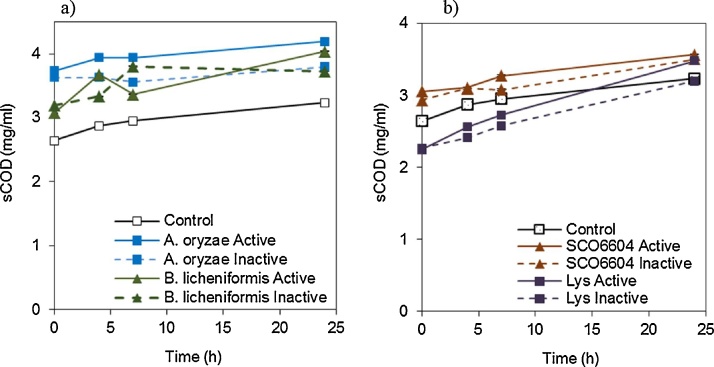
Soluble chemical oxygen demand (COD) content during enzymatic pretreatment of gamma irradiated biosludge for 24 h. a) Proteases and b) glycosidases. The control samples are biosludge with only phosphate buffer. Error bars (not always visible) represent the standard deviation of triplicates.

A possible mechanism for improved biogas production is additional COD solubilisation as a result of enzymatic pretreatment, making the COD more available for digestion. Soluble COD was measured and, as shown in Fig. 3, sCOD increased over time for all samples, including the controls with no enzyme, indicating that solubilisation was not specifically related to the pretreatment. Rather, many factors contribute to solubilisation, including gamma irradiation and residual enzymatic or microbial activity native to the sludge [[15], [26]]. Active and inactive enzymes showed similar trends over time and higher sCOD values do not correlate with higher biogas yields, suggesting that the positive effect in biogas production from the enzymes in this study was not the result of COD solubilisation. Previous reports show COD solubilisation as the mechanism for enhanced anaerobic digestibility [[6], [9]]. However, in this study, no evidence of COD solubilisation after enzymatic treatment was observed (Fig. 3a and b). Thus enhanced biogas production likely resulted from enzymatic reaction with substrates that were already present in the soluble portion of biosludge. It has to be noted that the initial sCOD of biosludge for BMP1 and 2 were 6.62 and 6.16 mg, respectively has a theoretical biogas potential of 3–3.75 mL. The success of the enzymatic pretreatment for enhancing anaerobic digestibility of biosludge can be affected by the limited conditions studied. It is conceivable that the enzymes used in this study, in particular the ones that did not show an increase in biogas production (BCE_2078 and CTec 2) or that showed marginal improvements (SCO6604 and lysozyme), could perform better under different conditions (e.g. enzyme dose, temperature, pH, time). Even the enzymes that showed a significant positive improvement might result in greater improvements under different conditions. Hence, to determine the maximum potential of the enzymes for increasing methane yields optimization of process variables is required. This would provide a more realistic idea of the potential of using enzymes for improving anaerobic digestion of biosludge.

Even though solubilization was not the main mechanism, the fact that enzymes improve the rate of biogas production and yields can lead to overall process improvements, potentially reducing the size of reactors as a result of reductions in residence times. Optimization of the treatment with the enzymes in this study and/or other enzymes may result in further biogas yield improvements. A dual treatment that first solubilizes COD and then uses enzymes to hydrolyze that soluble COD can improve the impact of enzymatic treatment on biosludge. Examples of these treatments could be thermal treatment and chemical treatments, such as surfactant addition which has shown to enhance the anaerobic digestibility of biosludge through the removal of EPS prior to enzymatic treatments [27].

### 3.4 Biogas production from enzyme solutions alone

**Fig. 4:**
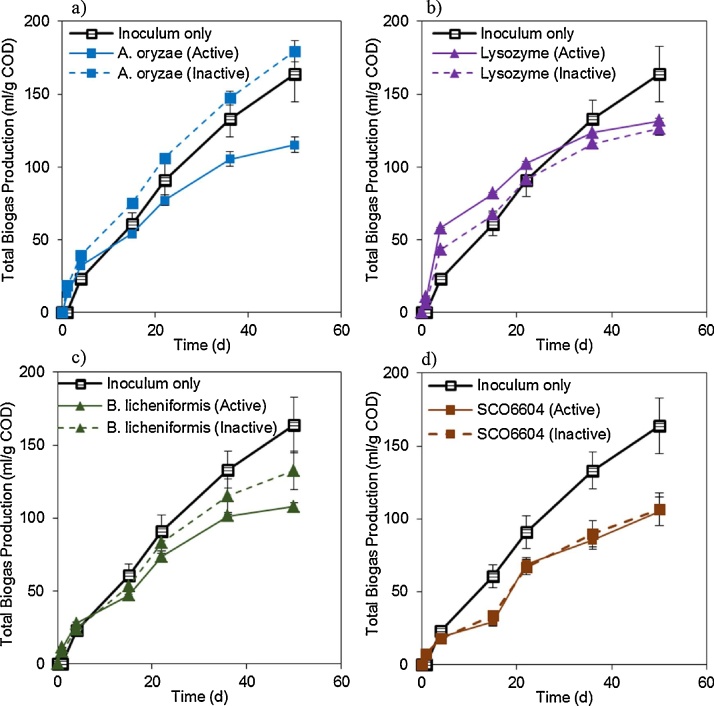
Biogas production from enzyme solutions. Total biogas productions (TBP) are presented for BMP 3, samples that contained enzyme solutions and inoculum. a) protease from *A. oryzae*; b) lysozyme; c) protease from *B. licheniformis*; d) glycosidase SCO6604. Inoculum only is the control, i.e. no enzyme added. Error bars show standard deviation of triplicates.

It is impossible to add enzyme without contributing a little to the total COD. The COD contributed by the enzyme solution alone was measured and in most cases, accounted for 2–5% of the total COD in the BMP assays, except for lysozyme that accounted for 13% of the total COD in BMPs. The same amounts were added to bottles with only inoculum and we found that this COD is not completely converted to biogas by the inoculum. Total biogas yields from these samples with enzyme and inoculum (no biosludge) from BMP 3 are shown in Fig. 4. The expectation was that samples with enzymes would produce more biogas than the “inoculum only” control because they contain higher COD (i.e. COD from the inoculum and COD from the enzyme solution); however, in most cases the opposite was observed (Fig. 4).

The COD in the enzyme solutions were not good biogas substrates (Fig. 4). In fact, in most cases, enzymes negatively affected the biogas yield of the inoculum and this effect was found to be enzyme-dependent. For example, samples containing active lysozyme resulted produced more biogas than the “inoculum only” control during the first 40 days, while samples containing protease from *B. licheniformis* produced the same or reduced biogas yield throughout the BMP assay (Fig. 4). The reasons for this are not quite clear. Perhaps enzyme solution components bind to the sludge EPS matrix and thus are not easily accessible for digestion. Or perhaps the enzyme activity (particularly in the case of lysozyme) decreases cell viability, thus, negatively affecting the inoculum activity. Overall, our results show that using theoretical biogas production based on the conversion of COD to CH_4_ to account for the effect of COD contributed by the enzyme is not recommended.

### 3.5 Potential of enzymatic activity assays to predict effect of enzymes on biosludge digestibility

**Fig. 5:**
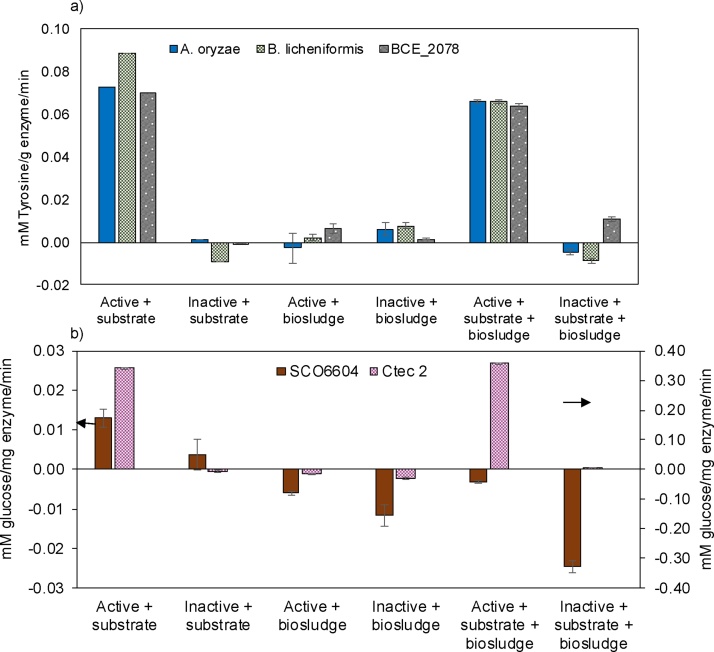
Enzymatic assays. a) protease activity assays for enzymes studied in BMP 1. Casein was used as the standard substrate. B) cellulose activity assays for enzymes studied in BMP 2 (except lysozyme). Casein was used as the standard substrate for proteases and CMC was used as the standard substrate for cellulases. Enzymatic activity was tested on standard substrates, biosludge and a combination. Active and inactive enzymes were included. Note the two vertical axis on b) are the same units but ranges are different. Error bars (not always visible) show standard deviation of triplicates.

Because BMP tests take a long time to complete, we wondered if potential enzymes could be pre-screened by running enzyme assays with biosludge itself as the substrate. To this end, enzymatic assays were performed using standard substrates, biosludge, and standard substrates added into biosludge (Fig. 5). The proteases from *B. licheniformis*, *A. oryzae*, and BCE_2078 showed enzymatic activities on casein as a standard substrate, as expected (Fig. 5a). These proteases showed very low enzymatic activities when provided with biosludge as the only substrate. BCE_2078 showed the highest activity in biosludge compared to the other proteases. Proteases in the presence of biosludge and casein showed significant enzymatic activity. This result indicates that the proteases were not inhibited or denatured by biosludge. Rather, the lower activity when compared to casein could suggest limited substrate (protein) availability in biosludge (Fig. 5a).

The results observed in these enzymatic assays did not correlate with the biogas yields obtained during BMP assays. Proteases from *A. oryzae* and BCE_2078 was most active in biosludge, but did not show any improvement in biogas production during BMP assays, while protease from *A. oryzae* showed significant potential for enhancing anaerobic digestion of biosludge. It is possible that while the proteases from *B. licheniformis* and *A. oryzae* found suitable substrates for hydrolysis in biosludge, BCE_2078 did not, thus, the difference in biogas production during the BMP assay. In addition, it is conceivable that the products of enzymatic hydrolysis are being consumed or transformed by the active microbial community in the BMP assays (i.e. biosludge and granules), and the net change during enzymatic treatment does not result in more easily digestible substrates, which could explain the lack of effect from BCE_2078 in BMP assays.

As seen in Fig. 5b, the glycosidases tested were active on the standard substrate (CMC). SCO6604 showed far less activity than CTec 2. When incubated with biosludge as a substrate, neither SCO6604 nor CTec 2 showed any activity. It is possible that because the assay relies on measuring the concentration of glucose released, microorganisms in biosludge consume the glucose and it cannot be measured by the colorimetric assay used in this study. When biosludge and CMC were provided together, only CTec 2 shows significant activity. However, biosludge pretreatment with CTec 2 did not show any significant increase in biogas during the BMP assays. SCO6604 showed no glycosidase activity in the presence of biosludge which suggests a possible inhibition or denaturation of the enzyme. The activity of lysozyme was measured using a standard substrate (*M. lysodeikticus* cells) and the inactivation was confirmed.

## 4 Conclusions

• Enzymes can enhance the anaerobic digestibility of biosludge as measured by BMP assays. In these tests, we found that the maximum improvement was 26% after 62 days of biosludge digestion when pretreated with the protease from *B. licheniformis*.
• All enzymes tested, except protease BCE_2078, were found to increase biogas production. These included the proteases from *B. licheniformis* and *A. oryzae*, a novel glycosidase (SCO6604), and lysozyme from chicken egg white.
• In order to determine the maximum potential of the enzymes in the improvement of anaerobic digestion and biogas production, working under optimal conditions is advised. Therefore, optimizing the enzymatic treatment conditions could be studied further.
• COD solubilisation could not be identified as the mechanism for enhancing anaerobic digestibility of biosludge in this study; therefore, it is suggested that a dual treatment that first solubilizes COD and then uses enzymes to hydrolyze the solubilized COD, may improve the impact of enzymatic treatment on biosludge.
• The COD of the enzyme solution is not 100% digestible to biogas; in some cases, enzyme solutions negatively affected the inoculum, and decreased biogas production.
• Enzymatic assays showed that enzymes retained their activity on standard substrates, even in biosludge, yet there was very low activity of the enzymes on biosludge but there was no significant inhibition or denaturation.
• No correlation was found between the enzymatic activities on standard substrates or biosludge, and the effect of enzymes on biogas production during BMP assays.
• A more rigorous approach for assessing impact of enzymatic treatment for enhanced anaerobic digestibility is proposed here, where the digestibility of the COD contributed by the enzyme solutions and the effect of enzymatic activity versus inactive enzymes are separately evaluated.
