# Supplementary material for: Evaluating the effect of enzymatic pretreatment on the anaerobic digestibility of pulp and paper biosludge
Source: Biotechnol Rep (Amst). 2018 Jan 5;17:77–85. doi: 10.1016/j.btre.2017.12.009 (PMC5767569; doi:10.1016/j.btre.2017.12.009)
Supplement: Supplementary file 1 [file mmc1.docx]

**Supplementary Material**

**Evaluating the effect of enzymatic pretreatment on the anaerobic digestibility of pulp and paper biosludge**

Sofia Bonilla^[[1]](#footnote-1)^, Zahra Choolaei^1^, Torsten Meyer, Elizabeth Edwards, Alexander F. Yakunin, D. Grant Allen^[[2]](#footnote-2)^,

Department of Chemical Engineering and Applied Chemistry at the University of Toronto, 200 College St., Toronto, Ontario M5S 3E5, Canada

Table of Contents

[Table S1. Biogas production for BMP 1 3](#_Toc463600743)

[Table S2. Biogas production for BMP 2 4](#_Toc463600744)

[Table S3. Biogas production for BMP 3 5](#_Toc463600745)

[Table S4. Specific biogas yield (SBY) for day 62 (final day) of BMP 1 6](#_Toc463600746)

[Table S5. Specific biogas yield for day 62 (final day) of BMP 2 6](#_Toc463600747)

[Table S6. Specific biogas yield (SBY) for day 50 (final day) of BMP 3 7](#_Toc463600748)

[Table S7. Total biogas production (TBP) for BMP 1 7](#_Toc463600749)

[Table S8. Total biogas production (TBP) for BMP 2. 8](#_Toc463600750)

[Table S9. Total biogas production (TBP) for BMP 3 9](#_Toc463600751)

[Table S10. Methane concentration (%) for BMP 1 bottles 10](#_Toc463600752)

[Table S11. Methane concentration (%) for BMP 2 bottles 10](#_Toc463600753)

[Table S12. Methane concentration (%) for BMP 3 bottles 11](#_Toc463600754)

[Table S13. Cumulative methane concentration (%) for BMP 1 bottles 12](#_Toc463600755)

[Table S14. Cumulative methane concentration (%) for BMP 2 bottles 13](#_Toc463600756)

[Table S15. Cumulative methane concentration (%) for BMP 3 bottles 13](#_Toc463600757)

[Table S16. Soluble chemical oxygen demand (sCOD) changes over the 24 h incubation period of enzymatic pretreatment of gamma irradiated biosludge 14](#_Toc463600758)

Table S1. Biogas production for BMP 1**, measured via the syringe method.**

| **BMP 1** | **Biogas production (ml), raw data** | | | | | | | | |
| --- | --- | --- | --- | --- | --- | --- | --- | --- | --- |
| **Time (day)** | **0** | **1** | **4** | **8** | **14** | **22** | **29** | **42** | **62** |
| **Biosludge only** | 0.0 | 4.9 ± 0.1 | 2.2 ± 0.3 | 2.3 ± 0.5 | 7.7 ± 0.5 | 5.4 ± 0.1 | 2.3 ± 0.2 | 5.5 ± 0.3 | 4.5 ± 0.2 |
| **Inoculum only** | 0.0 | 4.8 ± 0.4 | 1.2 ± 0.3 | 0.0 | 1.3 ±0.4 | 2.6 ± 0.1 | 0.7 ± 0.2 | 4.9 ± 0.1 | 2.4 ± 0.4 |
| **Positive control** | 0.0 | 41.2 ± 2.5 | 35.7 ± 0.6 | 7.8 ± 0.7 | 5.1 ± 0.1 | 4.1 ± 0.1 | 2.1 ± 0.6 | 4.9 ± 0.2 | 2.2 ± 0.3 |
| **Untreated (control)** | 0.0 | 6.2 ± 0.3 | 5.3 ± 0.3 | 2.7 ± 0.3 | 8.6 ± 0.3 | 6.1 ± 0.1 | 2.8 ± 0.3 | 8.5 ± 0.2 | 7.6 ± 0.5 |
| **Protease *A. oryzae* (Active)** | 0.0 | 7.9 ± 0.1 | 6.3 ± 0.1 | 5.3 ± 0.1 | 8.2 ± 0.4 | 6.1 ± 0.2 | 2.0 ± 0.1 | 7.3 ± 0.6 | 8.0 ± 0.5 |
| **Protease *A. oryzae* (Inactive)** | 0.0 | 7.4 ± 0.2 | 5.3 ± 0.2 | 4.6 ± 0.3 | 8.0 ± 0.2 | 5.6 ± 0.3 | 1.8 ± 0.3 | 7.4 ± 0.5 | 6.9 ± 0.4 |
| **Protease *B. licheniformis* (Active)** | 0.0 | 8.6 ± 0.1 | 6.8 ± 0.1 | 5.4 ± 0.5 | 6.6 ± 0.5 | 6.9 ± 0.2 | 2.0 ± 0.1 | 7.4 ± 0.8 | 6.9 ± 0.6 |
| **Protease *B. licheniformis* (Inactive)** | 0.0 | 6.8 ± 0.4 | 5.3 ± 0.2 | 4.7 ± 0.1 | 7.9 ± 0.2 | 3.8 ± 0.2 | 2.0 ± 0.2 | 8.2 ± 0.3 | 5.1 ± 0.2 |
| **Protease BCE2078 (Active)** | 0.0 | 7.1 ± 0.1 | 5.2 ± 0.3 | 5.1 ± 0.1 | 7.0 ± 0.4 | 5.3 ± 0.1 | 2.1 ± 0.1 | 8.2 ± 0.3 | 5.4 ± 0.1 |
| **Protease BCE2078 (Inactive)** | 0.0 | 7.1 ± 0.2 | 5.4 ± 0.4 | 4.3 ± 0.4 | 7.2 ± 0.3 | 6.2 ± 0.3 | 1.9 ± 0.1 | 7.2 ± 0.8 | 6.6 ± 0.6 |

Table S2. Biogas production for BMP 2**, measured via the syringe method.**

| **BMP 2** | **Raw biogas production (ml)** | | | | | | | | |
| --- | --- | --- | --- | --- | --- | --- | --- | --- | --- |
| **Time (day)** | **0** | **1** | **4** | **8** | **15** | **25** | **37** | **50** | **62** |
| **Biosludge only** | 0.0 | 3.7 ± 0.3 | 4.6 ± 0.3 | 2.9 ± 0.6 | 8.5 ± 0.9 | 5.7 ± 0.6 | 3.5 ± 0.3 | 2.5 ± 0.3 | 4.3 ± 0.3 |
| **Inoculum only** | 0.0 | 4.2 ± 0.2 | 2.3 ± 0.2 | 0.7 ± 0.2 | 1.1 ± 0.1 | 1.9 ± 0.1 | 0.8 ± 0.2 | 1.3 ± 0.6 | 4.0 ± 0.5 |
| **Positive Control** | 0.0 | 23.3 ± 1.5 | 41.0 ± 2.6 | 11.0 ± 0.7 | 3.9 ± 0.2 | 3.6 ± 0.3 | 1.3 ± 0.4 | 2.6 ± 0.3 | 3.0 ± 0.1 |
| **Untreated (control)** | 0.0 | 5.8 ± 0.1 | 6.7 ± 0.2 | 6.4 ± 0.2 | 7.9 ± 0.2 | 6.4 ± 0.3 | 4.8 ± 0.7 | 5.6 ± 0.3 | 6.5 ± 0.5 |
| **CTec 2(Active)** | 0.0 | 7.8 ± 0.7 | 9.3 ± 0.6 | 8.8 ± 0.5 | 11.6 ± 0.3 | 7.2 ± 0.1 | 5.0 ± 0.1 | 8.2 ± 0.3 | 5.9 ± 0.2 |
| **CTec 2 (Inactive)** | 0.0 | 8.2 ± 0.2 | 9.3 ± 0.3 | 9.0 ± 0.2 | 11.2 ± 0.4 | 6.6 ± 0.5 | 6.3 ± 0.4 | 6.1 ± 0.7 | 7.1 ± 0.4 |
| **Cellulase SCO6604 (Active)** | 0.0 | 6.8 ± 0.4 | 8.3 ± 0.7 | 8.2 ± 0.6 | 11.6 ± 0.3 | 4.3 ± 1.6 | 4.6 ± 0.4 | 8.2 ± 0.5 | 6.0 ± 0.3 |
| **Cellulase SCO6604 (Inactive)** | 0.0 | 6.9 ± 0.3 | 7.3 ± 0.1 | 7.2 ± 0.4 | 11.1 ± 0.6 | 4.2 ± 0.3 | 4.3 ± 0.2 | 8.6 ± 1.4 | 5.5 ± 0.5 |
| **Lysozyme (Active)** | 0.0 | 8.0 ± 0.4 | 13.9 ± 0.3 | 8.9 ± 0.3 | 11.4 ± 0.2 | 7.1 ± 0.4 | 4.0 ± 0.2 | 6.4 ± 0.6 | 4.9 ± 0.1 |
| **Lysozyme (Inactive)** | 0.0 | 7.3 ± 0.5 | 9.6 ± 0.1 | 10.8 ± 0.2 | 9.9 ± 0.1 | 6.9 ± 0.4 | 4.4 ± 0.4 | 7.8 ± 0.6 | 5.3 ± 0.8 |

Table S3. Biogas production for BMP 3**, measured via the syringe method.**

| **BMP 3** | **Biogas production (ml), raw data** | | | | | | | | |
| --- | --- | --- | --- | --- | --- | --- | --- | --- | --- |
| **Time (day)** | **0** | **1** | **4** | **8** | **15** | **22** | **29** | **36** | **50** |
| **Biosludge only** | 0 | 7.2 ± 0.1 | 1.1 ± 1 | 1.2 ± 0.3 | 0.9 ± 1 | 5.9 ± 3.8 | 2.2 ± 0.5 | 0.3 ± 0.6 | 1.5 ± 1.5 |
| **Inoculum only** | 0 | 8.5 | 3.5 ± 0.5 | 2.2 ± 0.6 | 2.9 ± 1 | 4.6 ± 0.5 | 3.3 ± 0.3 | 3.1 ± 1 | 4.6 ± 1.5 |
| **Positive control** | 0 | 44.6 ± 2.4 | 50.3 ± 1.6 | 11.2 | 5.0 ± 0.6 | 5.1 ± 0.2 | 3.2 ± 0.5 | 2.5 ± 1.1 | 4.8 ± 0.9 |
| **Untreated control** | 0 | 15.2 ± 0.3 | 14.8 ± 0.6 | 7.3 ± 0.6 | 6.2 ± 0.5 | 7.0 ± 0.5 | 5.3 ± 0.8 | 3.2 ± 0.5 | 5.7 ± 0.9 |
| **Protease *A. oryzae* (Active), no biosludge** | 0 | 10.5 ± 0.2 | 3.1 ± 0.1 | 0.0 | 3.5 ± 0.5 | 3.7 ± 0.5 | 0.0 | 4.5 ± 0.5 | 1.6 ± 0.4 |
| **Protease *A. oryzae* (Inactive), no biosludge** | 0 | 11.4 ± 0.1 | 3.4 ± 0.1 | 0.0 | 5.7 ± 0.4 | 5.0 ± 0.1 | 0.0 | 6.6 ± 1.1 | 5.2 ± 1.1 |
| **Protease *B. licheniformis* (Active), no biosludge** | 0 | 10.2 ± 0.1 | 2.6 ± 0.2 | 0.0 | 3.0 | 4.1± 0.4 | 0.0 | 4.3± 0.4 | 1.0± 1.1 |
| **Protease *B. licheniformis* (Inactive), no biosludge** | 0 | 9.8± 0.3 | 2.3± 0.3 | 0.0 | 4.6± 0.9 | 4.6± 0.6 | 0.0 | 5.0± 1.8 | 2.7± 0.5 |
| **Cellulase SCO6604 (Active), no biosludge** | 0 | 9.5± 0.5 | 1.9± 0.1 | 0.0 | 1.7± 0.6 | 6.1± 0.7 | 0.0 | 2.6± 0.6 | 3.1± 1 |
| **Cellulase SCO6604 (Inactive), no biosludge** | 0 | 9.6± 0.2 | 1.6± 0.6 | 0.0 | 2.4± 0.5 | 5.1± 0.8 | 0.0 | 3.5± 1.7 | 2.6± 1 |
| **Lysozyme (Active), no biosludge** | 0 | 10.3± 0.2 | 8.1± 0.2 | 0.0 | 4.1± 0.1 | 3.5± 0.5 | 0.0 | 3.6± 0.6 | 1.4± 0.6 |
| **Lysozyme (Inactive), no biosludge** | 0 | 9.6± 0.5 | 6.4± 0.4 | 0.0 | 4.2± 0.2 | 4.2± 0.4 | 0.0 | 4.3± 0.5 | 1.8± 0.2 |

Table S4. Specific biogas yield (SBY) for day 62 (final day) of BMP 1**. It was calculated as described in section 2.7.1 of the manuscript, via equation 1.**

| **BMP 1** | **SBY**  **(ml g ^-1^ COD fed)** |
| --- | --- |
| **Time (day)** | **62** |
| **Biosludge only** | 150 ± 4 |
| **Positive control** | 425 ± 11 |
| **Untreated (control)** | 149 ± 5 |
| **Protease *A. oryzae* (Active)** | 166 ± 5 |
| **Protease *A. oryzae* (Inactive)** | 145 ± 7 |
| **Protease *B. licheniformis* (Active)** | 163 ± 3 |
| **Protease *B. licheniformis* (Inactive)** | 130 ± 8 |
| **Protease BCE2078 (Active)** | 137 ± 2 |
| **Protease BCE2078 (Inactive)** | 140 ± 6 |

Table S5. Specific biogas yield for day 62 (final day) of BMP 2**. It was calculated as described in section 2.7.1 of the manuscript, via equation 1.**

| **BMP 2** | **SBY**  **(ml g ^-1^ COD fed)** |
| --- | --- |
| **Time (day)** | **62** |
| **Biosludge only** | 213 ± 4 |
| **Positive Control** | 489 ± 2 |
| **Untreated (control)** | 225 ± 7 |
| **CTec 2 (Active)** | 318 ± 4 |
| **CTec 2 (Inactive)** | 317 ± 9 |
| **Cellulase SCO6604 (Active)** | 277 ± 10 |
| **Cellulase SCO6604 (Inactive)** | 258 ± 3 |
| **Lysozyme (Active)** | 323 ± 6 |
| **Lysozyme (Inactive)** | 305 ± 9 |

Table S6. Specific biogas yield (SBY) for day 50 (final day) of BMP 3**. It was calculated as described in section 2.7.1 of the manuscript, via equation 1.**

| **BMP 3** | **SBY (ml g^-1^ COD fed)** |
| --- | --- |
| **Time (day)** | **50** |
| **Biosludge only** | 65 ± 30 |
| **Positive control** | 470 ± 20.5 |
| **Untreated control** | 160 ± 13.8 |

Table S7. Total biogas production (TBP) for BMP 1**. It was calculated as described in section 2.7.1 of the manuscript, via equation 2.**

| **BMP 1** | **TBP (ml g ^-1^ total COD)** | | | | | | | | |
| --- | --- | --- | --- | --- | --- | --- | --- | --- | --- |
| **Time (day)** | **0** | **1** | **4** | **8** | **14** | **22** | **29** | **42** | **62** |
| **Biosludge only** | 0 | 0 | 11 ± 4 | 23 ± 5 | 61 ± 8 | 88 ± 7 | 100 ± 9 | 127 ± 12 | 150 ± 10 |
| **Inoculum only** | 0 | -1 ± 1 | 12 ± 10 | 12 ± 10 | 28 ± 8 | 58 ± 9 | 67 ± 9 | 124 ± 10 | 152 ± 6 |
| **Positive control** | 0 | 127 ± 9 | 252 ± 7 | 279 ± 5 | 297 ± 5 | 311 ± 5 | 318 ± 7 | 335 ± 7 | 343 ± 8 |
| **Untreated (control)** | 0 | 5 ± 1 | 23 | 32 ± 1 | 63 ± 1 | 84 ± 1 | 94 ± 2 | 123 ± 1 | 150 ± 3 |
| **Protease *A. oryzae* (Active)** | 0 | 11 | 33 | 51 ± 1 | 80 ± 1 | 101 ± 1 | 108 ± 1 | 134 ± 2 | 162 ± 3 |
| **Protease *A. oryzae* (Inactive)** | 0 | 9 ± 1 | 27 ± 1 | 44 | 71 ± 1 | 91 ± 2 | 97 ± 2 | 123 ± 4 | 147 ± 5 |
| **Protease *B. licheniformis* (Active)** | 0 | 13 | 37 | 56 ± 2 | 79 ± 1 | 103 ± 2 | 110 ± 2 | 136 ± 2 | 160 ± 2 |
| **Protease *B. licheniformis* (Inactive)** | 0 | 7 ± 1 | 25 ± 1 | 42 ± 1 | 69 ± 1 | 83 ± 6 | 90 ± 6 | 118 ± 6 | 136 ± 6 |
| **Protease BCE2078 (Active)** | 0 | 8 | 26 ± 1 | 44 ± 1 | 68 ± 1 | 87 ± 1 | 94 ± 2 | 123 ± 1 | 142 ± 2 |
| **Protease BCE2078 (Inactive)** | 0 | 8 ± 1 | 27 | 42 ± 1 | 67 ± 2 | 89 ± 1 | 96 ± 2 | 121 ± 4 | 144 ± 4 |

Table S8. Total biogas production (TBP) for BMP 2. **It was calculated as described in section 2.7.1 of the manuscript, via equation 2.**

| **BMP 2** | **TBP (ml g^-1^ total COD)** | | | | | | | | |
| --- | --- | --- | --- | --- | --- | --- | --- | --- | --- |
| **Time (day)** | **0** | **1** | **4** | **8** | **15** | **25** | **37** | **50** | **62** |
| **Biosludge only** | 0 | 0 | 31 ± 2 | 50 ± 4 | 107 ± 2 | 145 ± 5 | 168 ± 3 | 185 ± 3 | 213 ± 4 |
| **Inoculum only** | 0 | 5 | 23 ± 2 | 29 ± 2 | 38 ± 2 | 53 ± 2 | 60 ± 3 | 70 ± 3 | 103 ± 3 |
| **Positive Control** | 0 | 72 ± 5 | 223 ± 5 | 263 ± 7 | 278 ± 8 | 291 ± 8 | 296 ± 8 | 305 ± 8 | 316 ± 8 |
| **Untreated (control)** | 0 | 8 ± 1 | 33 ± 1 | 56 ± 1 | 85 ± 1 | 109 ± 2 | 126 ± 3 | 147 ± 3 | 171 ± 4 |
| **CTec 2 (Active)** | 0 | 15 ± 2 | 50 ± 1 | 82 ± 2 | 124 ± 2 | 151 ± 2 | 169 ± 2 | 200 ± 2 | 221 ± 2 |
| **CTec 2 (Inactive)** | 0 | 17 | 51 ± 2 | 84 ± 2 | 125 ± 2 | 149 ± 3 | 173 ± 4 | 195 ± 4 | 221 ± 5 |
| **Cellulase SCO6604 (Active)** | 0 | 11 ± 1 | 42 ± 2 | 72 ± 4 | 114 ± 4 | 130 ± 4 | 147 ± 6 | 177 ± 6 | 199 ± 5 |
| **Cellulase SCO6604 (Inactive)** | 0 | 12 ± 1 | 39 ± 2 | 65 ± 1 | 106 ± 2 | 121 ± 2 | 137 ± 3 | 169 ± 3 | 189 ± 2 |
| **Lysozyme (Active)** | 0 | 16 ± 1 | 67 ± 2 | 100 ± 2 | 142 ± 1 | 168 ± 2 | 183 ± 3 | 206 ± 3 | 225 ± 3 |
| **Lysozyme (Inactive)** | 0 | 13 ± 2 | 49 ± 2 | 88 ± 2 | 125 ± 3 | 150 ± 4 | 166 ± 6 | 195 ± 6 | 215 ± 5 |

Table S9. Total biogas production (TBP) for BMP 3**. It was calculated as described in section 2.7.1 of the manuscript, via equation 2.**

| **BMP 3** | **TBP (ml g^-1^ total COD)** | | | | | | | | |
| --- | --- | --- | --- | --- | --- | --- | --- | --- | --- |
| **Time (day)** | **0** | **1** | **4** | **8** | **15** | **22** | **29** | **36** | **50** |
| **Biosludge only** | 0 | 0 ± 0.3 | 6 ± 4.5 | 12 ± 2.8 | 16 ± 4.7 | 46 ± 20.9 | 56 ± 22.0 | 58 ± 24.6 | 65 ± 30.0 |
| **Inoculum only** | 0 | 8 | 31 ± 2.5 | 46 ± 5.6 | 65 ± 5.8 | 96 ± 6.5 | 117 ± 8.6 | 138 ± 8.2 | 168 ± 12.1 |
| **Positive control** | 0 | 106 ± 6.7 | 250 ± 3.7 | 281 ± 3.8 | 296 ± 4.2 | 310 ± 4.5 | 319 ± 6.7 | 326 ± 9.2 | 340 ± 11.7 |
| **Untreated control** | 0 | 23 ± 0.8 | 65 ± 2.2 | 86 ± 3.1 | 103 ± 3.5 | 123 ± 4.7 | 138 ± 6.7 | 147 ± 5.3 | 164 ± 7.9 |
| **Protease *A. oryzae* (Active), no biosludge** | 0 | 21 ± 1.0 | 40 ± 1.1 | 40 ± 1.1 | 62 ± 4.1 | 85 ± 7.0 | 85 ± 7.0 | 113 ± 9.6 | 123 ± 10.2 |
| **Protease A. *oryzae* (Inactive), no biosludge** | 0 | 26 ± 0.7 | 47 ± 0.9 | 47 ± 0.9 | 83 ± 1.6 | 114 ± 1.9 | 114 ± 1.9 | 155 ± 7.9 | 187 ± 12.8 |
| **Protease *B. licheniformis* (Active), no biosludge** | 0 | 19 ± 0.6 | 36 ± 1.3 | 36 ± 1.3 | 55 ± 1.3 | 82 ± 3.9 | 82 ± 3.9 | 109 ± 2.3 | 116 ± 4.5 |
| **Protease *B. licheniformis* (Inactive), no biosludge** | 0 | 17 ± 2 | 32 ± 2.4 | 32 ± 2.4 | 61 ± 7.1 | 91 ± 10.5 | 91 ± 10.5 | 123 ± 22 | 141 ± 25.1 |
| **Cellulase SCO6604 (Active), no biosludge** | 0 | 14 ± 3 | 27 ± 3 | 27 ± 3 | 38 ± 5.4 | 77 ± 8.8 | 77 ± 8.8 | 93 ± 11.8 | 113 ± 18.6 |
| **Cellulase SCO6604 (Inactive), no biosludge** | 0 | 16 ± 1.5 | 26 ± 2.6 | 26 ± 2.6 | 425.4 | 75 ± 10.2 | 75 ± 10.2 | 98 ± 17.5 | 115 ± 22.1 |
| **Lysozyme (Active), no biosludge** | 0 | 18 ± 1.0 | 65 ± 1.0 | 65 ± 1.0 | 89 ± 0.9 | 110 ± 2.8 | 110 ± 2.8 | 131 ± 0.6 | 139 ± 3.8 |
| **Lysozyme (Inactive), no biosludge** | 0 | 13 ± 2.8 | 50 ± 3.2 | 50 ± 3.2 | 75 ± 7.0 | 99 ± 10.1 | 99 ± 10.1 | 123 ± 20.6 | 134 ± 23.5 |

Table S10. Methane concentration (%) for BMP 1 bottles**, measured using gas chromatography (GC).**

| **BMP 1** | **Methane concentration (%), from GC measurements** | | | | | | | | |
| --- | --- | --- | --- | --- | --- | --- | --- | --- | --- |
| **Time (day)** | **0** | **1** | **4** | **8** | **14** | **22** | **29** | **42** | **62** |
| **Biosludge only** | 0.0% | 0.0% | 1.3% | 0.2% | 12.2% | 16.9% | 20.1% | 22.0% | 26.3% |
| **Inoculum only** | 0.0% | 0.0% | 1.7% | 0.2% | 0.2% | 8.5% | 10.9% | 13.2% | 16.6% |
| **Positive control** | 0.0% | 23.6% | 43.0% | 45.0% | 40.8% | 46.7% | 51.7% | 47.4% | 52.3% |
| **Untreated (control)** | 0.0% | 2.1% | 6.4% | 11.9% | 17.1% | 21.2% | 25.7% | 26.9% | 33.8% |
| **Protease *A. oryzae* (Active)** | 0.0% | 3.4% | 8.5% | 14.3% | 20.7% | 23.9% | 27.8% | 31.1% | 35.2% |
| **Protease *A. oryzae* (Inactive)** | 0.0% | 3.0% | 7.4% | 13.9% | 18.4% | 22.8% | 25.4% | 27.1% | 33.5% |
| **Protease *B. licheniformis* (Active)** | 0.0% | 4.3% | 10.4% | 15.3% | 20.4% | 24.5% | 28.1% | 31.3% | 36.0% |
| **Protease *B. licheniformis* (Inactive)** | 0.0% | 2.6% | 7.1% | 13.0% | 18.5% | 21.4% | 25.2% | 28.6% | 32.9% |
| **Protease BCE2078 (Active)** | 0.0% | 2.2% | 6.7% | 13.0% | 18.3% | 20.7% | 25.6% | 27.8% | 32.8% |
| **Protease BCE2078 (Inactive)** | 0.0% | 2.0% | 7.4% | 12.9% | 18.9% | 22.6% | 24.9% | 28.2% | 32.1% |

Table S11. Methane concentration (%) for BMP 2 bottles**, measured using gas chromatography (GC).**

| **BMP 2** | **Methane concentration (%) from GC** | | | | | | |
| --- | --- | --- | --- | --- | --- | --- | --- |
| **Time (day)** | **0** | **1** | **4** | **8** | **15** | **50** | **62** |
| **Biosludge only** | 0.0% | 0.2% | 0.2% | 4.5% | 12.7% | 23.4% | 24.8% |
| **Inoculum only** | 0.0% | 0.2% | 0.2% | 1.8% | 0.2% | 9.8% | 12.5% |
| **Positive Control** | 0.0% | 12.6% | 37.1% | 41.3% | 40.6% | 46.0% | 46.6% |
| **Untreated (control)** | 0.0% | 2.4% | 5.5% | 10.6% | 16.7% | 30.3% | 32.2% |
| **CTec 2(Active)** | 0.0% | 4.0% | 8.8% | 16.5% | 22.5% | 36.3% | 38.0% |
| **CTec 2 (Inactive)** | 0.0% | 4.0% | 9.0% | 16.7% | 21.4% | 36.7% | 38.4% |
| **Cellulase SCO6604 (Active)** | 0.0% | 4.1% | 7.5% | 14.9% | 22.0% | 33.7% | 35.7% |
| **Cellulase SCO6604 (Inactive)** | 0.0% | 2.7% | 6.8% | 14.5% | 18.7% | 32.4% | 34.3% |
| **Lysozyme (Active)** | 0.0% | 3.1% | 13.4% | 20.0% | 23.7% | 36.4% | 37.7% |
| **Lysozyme (Inactive)** | 0.0% | 3.8% | 9.6% | 18.4% | 22.7% | 35.5% | 36.8% |

Table S12. Methane concentration (%) for BMP 3 bottles**, measured using gas chromatography (GC).**

| **BMP 3** | **Methane concentration (%) from GC** | | | | |
| --- | --- | --- | --- | --- | --- |
| **Time (day)** | **0** | **1** | **4** | **15** | **50** |
| **Biosludge only** | 0.0% | 0.2% | 0.2% | 2.2% | 0.2% |
| **Inoculum only** | 0.0% | 1.3% | 4.4% | 9.8% | 27.0% |
| **Positive control** | 0.0% | 26.3% | 56.3% | 50.9% | 65.7% |
| **Untreated control** | 0.0% | 9.7% | 18.4% | 24.1% | 41.0% |
| **Protease *A. oryzae* (Active), no biosludge** | 0.0% | 2.3% | 5.5% | 8.8% | 18.4% |
| **Protease *A. oryzae* (Inactive), no biosludge** | 0.0% | 2.8% | 6.1% | 12.5% | 28.1% |
| **Protease *B. licheniformis* (Active), no biosludge** | 0.0% | 2.3% | 5.1% | 8.4% | 19.4% |
| **Protease *B. licheniformis* (Inactive), no biosludge** | 0.0% | 2.2% | 4.1% | 10.4% | 24.4% |
| **Cellulase SCO6604 (Active), no biosludge** | 0.0% | 1.5% | 3.5% | 8.2% | 17.6% |
| **Cellulase SCO6604 (Inactive), no biosludge** | 0.0% | 0.2% | 3.8% | 5.9% | 17.4% |
| **Lysozyme (Active), no biosludge** | 0.0% | 1.9% | 10.0% | 13.5% | 24.7% |
| **Lysozyme (Inactive), no biosludge** | 0.0% | 1.5% | 8.8% | 12.4% | 21.8% |

Table S13. Cumulative methane concentration (%) for BMP 1 bottles**.**

| **BMP 1** | **Cumulative methane concentration(%) *** | | | | | | | | |
| --- | --- | --- | --- | --- | --- | --- | --- | --- | --- |
| **Time (day)** | **0** | **1** | **4** | **8** | **14** | **22** | **29** | **42** | **62** |
| **Biosludge only** | 0% | 0% | 14% | 2% | 60% | 66% | 71% | 67% | 71% |
| **Inoculum only** | 0% | 0% | 22% | 3% | 2% | 65% | 77% | 69% | 76% |
| **Positive control** | 0% | 69% | 77% | 76% | 70% | 74% | 78% | 73% | 76% |
| **Untreated (control)** | 0% | 28% | 47% | 70% | 68% | 69% | 76% | 68% | 74% |
| **Protease *A. oryzae* (Active)** | 0% | 37% | 52% | 65% | 70% | 69% | 75% | 73% | 74% |
| **Protease *A. oryzae* (Inactive)** | 0% | 34% | 50% | 69% | 67% | 70% | 74% | 68% | 74% |
| **Protease *B. licheniformis* (Active)** | 0% | 44% | 60% | 66% | 70% | 70% | 75% | 73% | 76% |
| **Protease *B. licheniformis* (Inactive)** | 0% | 32% | 50% | 67% | 69% | 70% | 77% | 73% | 76% |
| **Protease BCE2078 (Active)** | 0% | 26% | 46% | 65% | 68% | 66% | 75% | 70% | 74% |
| **Protease BCE2078 (Inactive)** | 0% | 24% | 50% | 66% | 71% | 71% | 73% | 72% | 72% |

* Cumulative methane concentration was calculated through the following steps:

$$Gas volume on sampling day \left( ml \right)=Raw biogas production \left( ml \right)+0.2 ml GC sampling volume+80 ml bottle head space$$

$${CH}_{4} volume on sampling day (ml)=\frac{Gas volume on sampling day \left( ml \right)\times{CH}_{4} concentration from GC (\%)}{100}$$

$${CH}_{4} production on sampling day \left( ml \right)={CH}_{4} volume on sampling day-\frac{80 ml head space\times Previous sampling day^{'}s {CH}_{4} \% from GC}{100}$$

$$Cumulative {CH}_{4} concentration \left( \% \right)=\frac{Cumulative {CH}_{4}(ml)}{Cumulative biogas (ml)}\times100$$

Table S14. Cumulative methane concentration (%) for BMP 2 bottles**.**

| **BMP 2** | **Cumulative methane concentration (%) *** | | | | | | |
| --- | --- | --- | --- | --- | --- | --- | --- |
| **Time (day)** | **0** | **1** | **4** | **8** | **15** | **50** | **62** |
| **Biosludge only** | 0% | 4% | 2% | 32% | 56% | 80% | 76% |
| **Inoculum only** | 0% | 4% | 3% | 19% | 2% | 71% | 69% |
| **Positive Control** | 0% | 55% | 74% | 74% | 71% | 75% | 75% |
| **Untreated (control)** | 0% | 34% | 38% | 50% | 58% | 79% | 75% |
| **CTec 2(Active)** | 0% | 44% | 47% | 60% | 61% | 76% | 74% |
| **CTec 2 (Inactive)** | 0% | 42% | 47% | 59% | 58% | 77% | 74% |
| **Cellulase SCO6604 (Active)** | 0% | 51% | 45% | 59% | 63% | 74% | 73% |
| **Cellulase SCO6604 (Inactive)** | 0% | 33% | 42% | 61% | 57% | 73% | 72% |
| **Lysozyme (Active)** | 0% | 33% | 58% | 64% | 60% | 74% | 73% |
| **Lysozyme (Inactive)** | 0% | 45% | 51% | 63% | 62% | 75% | 74% |

Table S15. Cumulative methane concentration (%) for BMP 3 bottles**.**

| **BMP 3** | **Cumulative methane (%) concentration** | | | | |
| --- | --- | --- | --- | --- | --- |
| **Time (day)** | **0** | **1** | **4** | **15** | **50** |
| **Biosludge only** | 0% | 3% | 2% | 16% | 1% |
| **Inoculum only** | 0% | 13% | 31% | 49% | 80% |
| **Positive control** | 0% | 73% | 90% | 80% | 87% |
| **Untreated control** | 0% | 60% | 63% | 61% | 75% |
| **Protease *A. oryzae* (Active), no biosludge** | 0% | 21% | 40% | 55% | 68% |
| **Protease *A. oryzae* (Inactive), no biosludge** | 0% | 25% | 45% | 75% | 102% |
| **Protease *B. licheniformis* (Active), no biosludge** | 0% | 20% | 25% | 34% | 57% |
| **Protease *B. licheniformis* (Inactive), no biosludge** | 0% | 21% | 23% | 45% | 74% |
| **Cellulase SCO6604 (Active), no biosludge** | 0% | 12% | 22% | 40% | 59% |
| **Cellulase SCO6604 (Inactive), no biosludge** | 0% | 2% | 21% | 25% | 46% |
| **Lysozyme (Active), no biosludge** | 0% | 17% | 64% | 71% | 88% |
| **Lysozyme (Inactive), no biosludge** | 0% | 14% | 59% | 63% | 71% |

Table S16. Soluble chemical oxygen demand (sCOD) changes over the 24 h incubation period of enzymatic pretreatment of gamma irradiated biosludge**.**

| **Sample ID** | **Δ sCOD (mg/ml)** |
| --- | --- |
| **Control** | 0.6 |
| ***A. oryzae* Active** | 0.5 |
| ***A. oryzae* Inactive** | 0.2 |
| ***B. licheniformis* Active** | 1.0 |
| ***B. licheniformis* Inactive** | 0.5 |
| **SCO6604 Active** | 0.5 |
| **SCO6604 Inactive** | 0.6 |
| **Lysozyme Active** | 1.2 |
| **Lysozyme Inactive** | 0.9 |

1. these authors contributed equally to this work. [↑](#footnote-ref-1)
2. Corresponding author [↑](#footnote-ref-2)
